# Exciton-dielectric mode coupling in MoS_2_ nanoflakes visualized by cathodoluminescence

**DOI:** 10.1515/nanoph-2021-0643

**Published:** 2022-01-12

**Authors:** Dung Thi Vu, Nikolaos Matthaiakakis, Hikaru Saito, Takumi Sannomiya

**Affiliations:** Department of Materials Science and Engineering, School of Materials and Chemical Technology, Tokyo Institute of Technology, 4259 Nagatsuta Midoriku, Yokohama 226-8503, Japan; Theoretical and Physical Chemistry Institute, National Hellenic Research Foundation, NHRF, 48 Vassileos Constantinou Ave., 11635 Athens, Greece; Institute for Materials Chemistry and Engineering, Kyushu University, Fukuoka 816-8580, Japan; Pan-Omics Data-Driven Research Innovation Center, Kyushu University, Fukuoka 816-8580, Japan

**Keywords:** cathodoluminescence, exciton-dielectric mode coupling, localized field, molybdenum disulfide, scanning transmission electron microscopy

## Abstract

Two-dimensional (2D) transition metal dichalcogenides (TMDCs), possessing unique exciton luminescence properties, have attracted significant attention for use in optical and electrical devices. TMDCs are also high refractive index materials that can strongly confine the electromagnetic field in nanoscale dimensions when patterned into nanostructures, thus resulting in complex light emission that includes exciton and dielectric resonances. Here, we use cathodoluminescence (CL) to experimentally visualize the emission modes of single molybdenum disulfide (MoS_2_) nanoflakes and to investigate luminescence enhancement due to dielectric resonances in nanoscale dimensions, by using a scanning transmission electron microscope. Specifically, we identify dielectric modes whose resonant wavelength is sensitive to the shape and size of the nanoflake, and exciton emission peaks whose energies are insensitive to the geometry of the flakes. Using a four-dimensional CL method and boundary element method simulations, we further theoretically and experimentally visualize the emission polarization and angular emission patterns, revealing the coupling of the exciton and dielectric resonant modes. Such nanoscopic observation provides a detailed understanding of the optical responses of MoS_2_ including modal couplings of excitons and dielectric resonances which play a crucial role in the development of energy conversion devices, single-photon emitters, and nanophotonic circuits with enhanced light-matter interactions.

## 1 Introduction

Molybdenum disulfide (MoS_2_), one of the most common materials amongst two-dimensional (2D) transition metal dichalcogenides (TMDCs) [1, 2], has received significant attention owing to its semiconductive nature, tunable bandgap, and its strong light-absorption capacity in the visible region. These properties enable its implementation in the production of semiconductor sensors [3], photocatalytic [4], hydrothermal and optoelectronic devices [2]. The indirect bandgap of bulk MoS_2_ semiconductor switches to a direct bandgap when its thickness is reduced to a monolayer, thus providing a surprisingly stronger luminescence than bulk MoS_2_ [5]. Materials of the TMDC family, with a general formula of MX_2_ (where M = Mo, W; X = S, Se, Te) possess high refractive indices [4], [5], [6], [7], [8], [9] and are capable of optical field confinement that can enhance light-matter interactions based on the formation of dielectric cavity modes [10, 11]. It has been demonstrated that WS_2_ nanodisks fabricated from multilayer WS_2_ possess characteristics that allow coupling between Mie resonant modes excited in nanostructures that are sensitive to geometrical characteristics, and A-excitons originating from the intrinsic material properties of WS_2_ [4]. In this study, we are interested in the intrinsic luminescence characteristics and resonant modes occurring in single MoS_2_ nanoflakes that emit light in the visible spectral range. To probe and understand the origin of the sub-wavelength optical properties of the MoS_2_ nanostructures, visualization of the optical properties beyond the diffraction limit of light is required, especially for the identification of the distinct mechanisms behind the multiple types of light emission [12, 13]. This can be achieved by using an electron beam as a high-energy and high-momentum excitation source with a size of down to 1 nm, thus leading to the excitation of all electron transitions in the material with nanoscale resolution, something not possible with photoluminescence spectroscopy [13], [14], [15]. However, there is currently no nanoscopic measurement available in the literature investigating the optical response of TMDCs at nanoscale dimensions.

**Figure 1:**
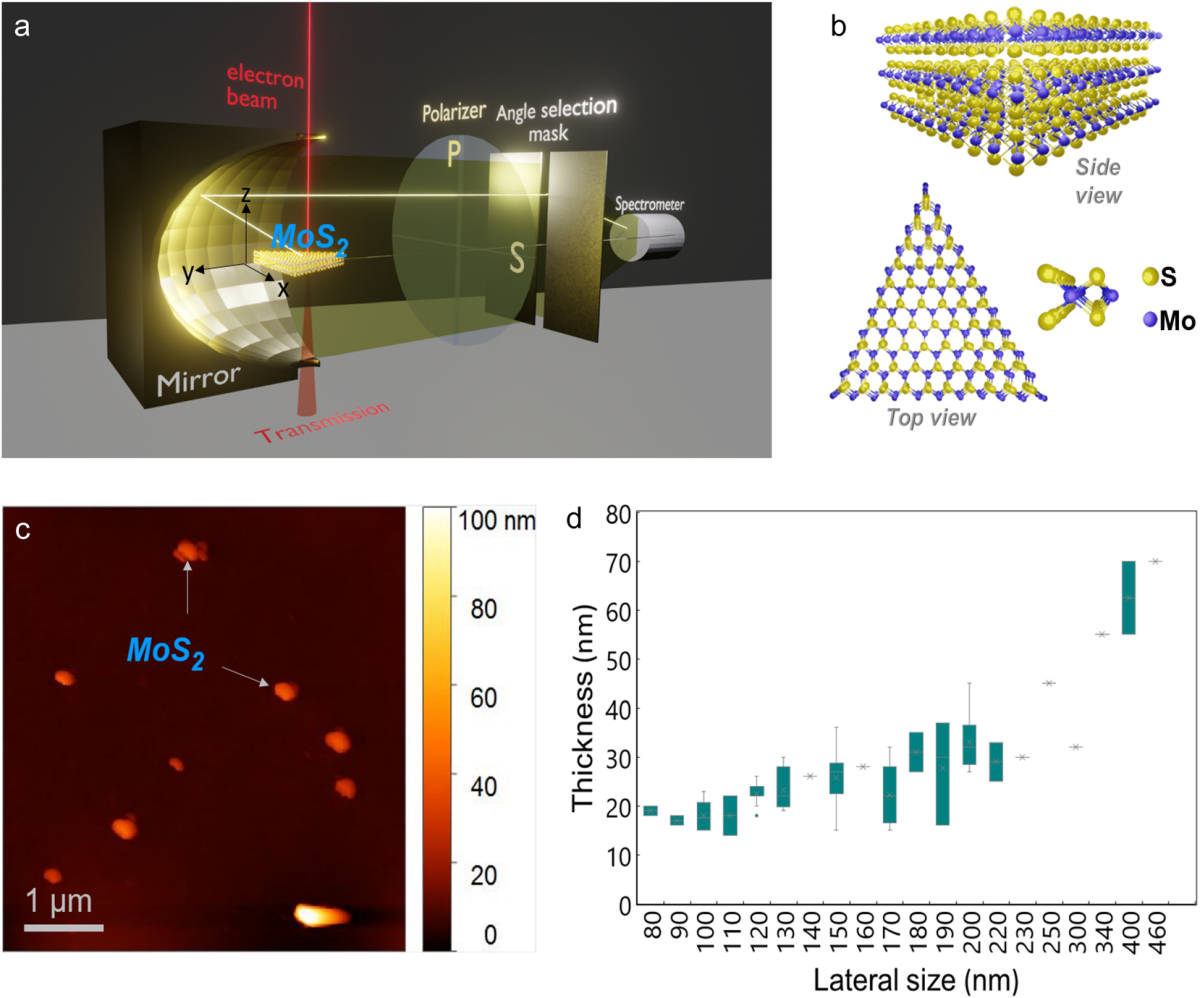
Experimental CL-STEM setup and sample morphology. (a) Schematic illustration of STEM-CL system. (b) The crystal structure of a triangular multiple-layer MoS_2_. In monolayer MoS_2_, molybdenum atoms (blue) are sandwiched between two layers of sulfur atoms (yellow). (c) AFM image of MoS_2_ flakes on a flat substrate. (d) Lateral size and thickness relation of the flakes measured by AFM.

Cathodoluminescence (CL) has been used to study the composition and defects of material structures and is also widely utilized to study the effect of dopants in insulators and semiconductors as well as for the study of optical modes in nanophotonics [16, 17]. Thus, we believe that CL is a powerful approach for accessing the nanoscopic optical information of MoS_2_. Here in this study, we experimentally visualize emission processes supported in isolated MoS_2_ nanoflakes through CL mapping, by using a CL–STEM system as described in Figure 1a. Firstly, we map the photon emission distribution on individual nanoflakes, as well as compare emission wavelengths for various flake shapes. The obtained CL mapping data is spectrally deconvoluted in order to separate the overlapped emission modes of the nanoflake and the dielectric mode is further identified based on the field distribution. Based on the identified deconvoluted peaks, we discuss how the resonant modes contribute to luminescence enhancement. In addition, since measured CL signals consist of superpositions of multiple emission modes, we employ the four-dimensional (4D) CL technique [18] that allows us to obtain the angular spectrum at excited beam positions, and extract photon energy maps in all radiation angles from the flake.

## 2 Methods

### 2.1 Cathodoluminescence measurement

The CL system is based on a JEM-2100F STEM (JEOL Ltd.) operated at 80 kV electron acceleration voltage achieving a 1 nm spatial resolution with 1 nA beam current [19, 20]. As shown in Figure 1a, an aluminum parabolic mirror is installed inside the STEM vacuum chamber to collimate the radiation emitted from the specimen placed inside. The collimated radiation then passes through an objective lens, polarizers, or mask system to be detected by the spectrometer. In the 4D CL system [18], a slit mask is inserted in the radiation path parallel to the *z*-axis, behind the objective lens, polarizers and masks. The radiation is transferred through the optical system to the spectrometer with the information on the emission angle dependence projected along the vertical axis. Therefore, by 4D STEM-CL, the angular distribution and spectrum at each beam position are obtained simultaneously. To obtain a CL photon map, while the electron beam scans over the specimen, the CL signals generated through optical excitation by fast electrons are collected at each electron beam position. However, in the raw spectra from the CL photon map data, the broad overlapping peaks had been difficult to be identified. Here, we conducted a spectral deconvolution of the CL photon map data using Lorentzian fitting to present the individual energy modes in the MoS_2_ flakes. The TEM specimen was prepared by depositing the MoS_2_ flake solution onto an elastic carbon grid (Okenshoji Co., Ltd., Japan).

### 2.2 Boundary element method simulation

We simulate cathodoluminescence emitted from MoS_2_ nanoflakes using boundary element method (BEM) simulations within the MNPBEM toolbox [21], [22], [23]. The BEM simulations are based on the calculation of spectra using Maxwell’s equations in the frequency domain. The simulation CL signal is produced by an electron beam with 80 kV electron acceleration voltage to be consistent with CL measurement conditions. The simulation also allows resolving the CL radiation signals to be resolved in polarization and angle of detection by angularly rotating the detector around the structure.

## 3 Results and discussion

### 3.1 Emission modes of single MoS_2_ nanoflakes

**Figure 2:**
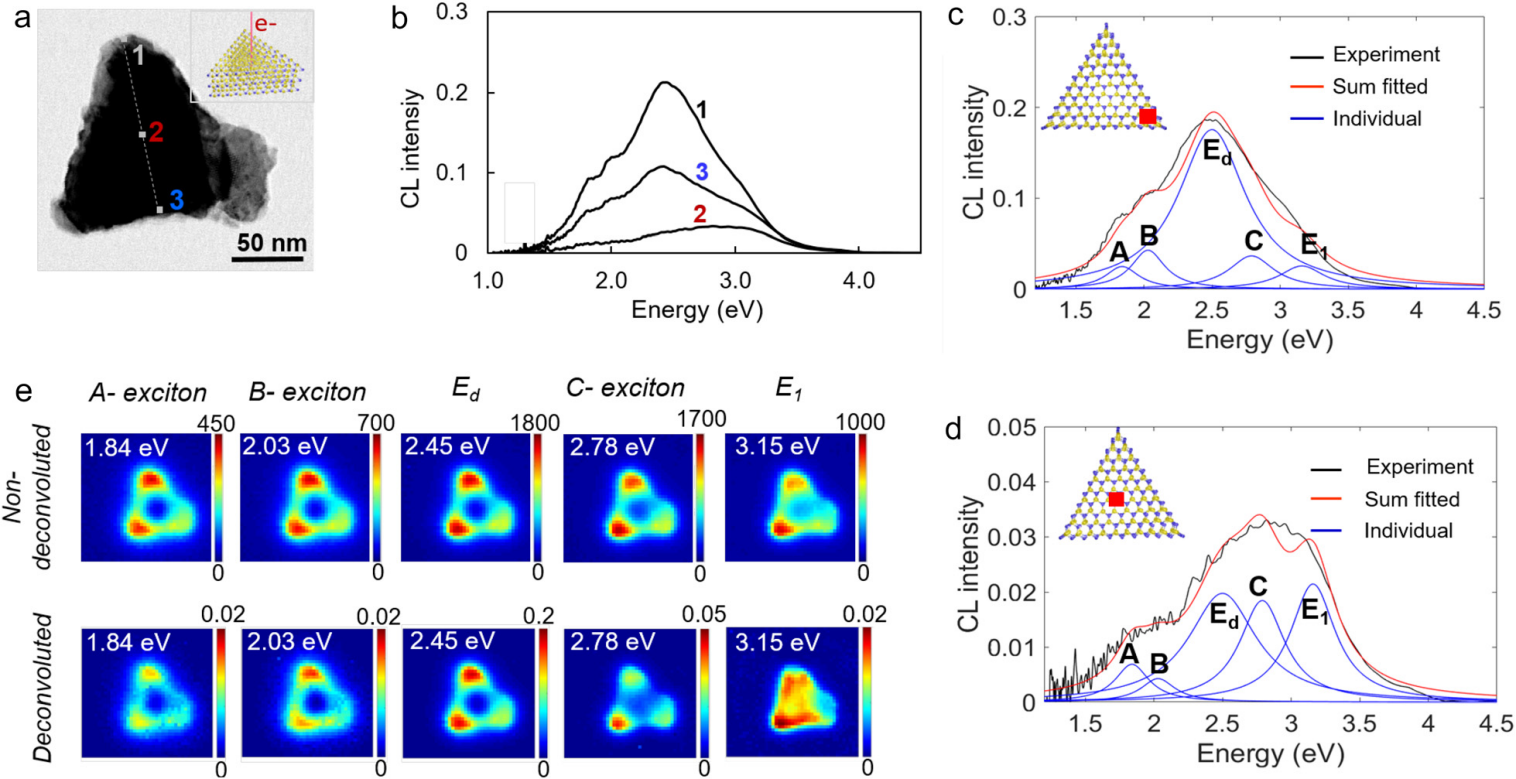
Mode analysis of triangular nanoflake. (a) STEM bright field image of a single MoS_2_ particle. The insert is an illustration of the electron beam impinging on the triangular MoS_2_ nanoflake. (b) Line-scan CL spectra along electron beam positions 1–3 as shown in Figure 2a. (c) and (d) The spectra obtained for excitation at the edge and the center of the flake respectively. The spectral deconvolution is performed by fitting the experimental data (black line) to a sum of individual Lorentzian curves (blue lines). The peak positions of the individual curves are 1.84 eV (A-exciton), 2.03 eV (B-exciton), 2.45 eV (*E*_*d*_), 2.78 eV (C-exciton), and 3.15 eV (*E*_1_). (e) Comparison of non-polarized CL maps with and without spectra deconvolution at 1.84 eV (A-exciton), 2.03 eV (B-exciton), 2.45 eV (*E*_*d*_), 2.78 eV (C-exciton), and 3.15 eV (*E*_1_), according to individual peaks shown in Figure 2c and d. Mapping data is obtained from the same flake shown in Figure 2a. The CL signals are collected in all the emission angles without polarization selectivity.

In this section, we identify the optical modes of the MoS_2_ by collecting radiated emission from the entire range of emission angles supported by the parabolic mirror (Figure 1a). The crystal structure of a triangular flake is illustrated in Figure 1b, which is responsible for the typical flat and thin structure for the small flakes. Figure 1c shows the atomic force microscopy (AFM) image of the used MoS_2_ flakes (Nanoflake solution, EM-Japan Co. Ltd., Japan) on a flat substrate. The relation between the thickness and lateral dimension can be obtained from the AFM images of various flakes as shown in Figure 1d. Figure 2a shows the bright-field STEM image of a triangular MoS_2_ flake with a lateral size of 160 nm and a corresponding thickness of approximately 30 nm according to Figure 1d. Figure 2b shows line-scan spectra obtained through positioning the electron beam along positions 1–3 of the flake as labelled in Figure 2a. The spectra significantly depend on the excitation position and have a higher CL intensity at position 1 compared to positions 2 and 3. The CL spectra of the nanoflake consist of multiple overlapping energy peaks, thus in order to better identify the peaks they are fitted with a multiple peak model that corresponds to the known optical modes of MoS_2_. Figure 2c and d shows multiple peak fitting for the spectra obtained at the 1 and 2 positions respectively. In this, the A peak at 1.84 eV and the B peak at 2.03 eV are attributed to the A-exciton and B-exciton, that occur due to a direct and indirect bandgap, respectively [5, 24, 25]. The C peak at 2.78 eV is associated with the C-exciton, originating from transitions between the highest valence band and the first three lowest conduction bands of MoS_2_ [26, 27]. As reported by V.L. Le [28], the *E*_1_ peak at 3.15 eV can be attributed to the exciton transition band at the M point of the MoS_2_ band structure. We also observe a prominent peak at 2.5 eV (*E*_*d*_) that does not correspond to any known exciton transitions of MoS_2_, and has a strong dependence on excitation position. As will be discussed later, the energy of the *E*_*d*_ peak depends on the shape and size of the flake, and thus *E*_*d*_ can be attributed to a geometrically dependent dielectric resonant mode that is excited due to the high refractive index of MoS_2_ [4]. Multiple dielectric modes can also be supported by one structure if the flake supports nondegenerate dielectric modes. We further observe that the exciton peaks A and B become higher for edge excitation, rather than for excitation at the center, in spite of the almost uniform thickness of the flake, suggesting the existence of a luminescence enhancement mechanism at the edges.

Figure 2e shows nondeconvoluted and deconvoluted CL maps by multiple peak fitting selected at the mode energies of the same flake of Figure 2a. For deconvoluted CL maps, the intensity of each emission peak is presented separately, excluding overlapping spectra from nearby peaks. In the fitting process for deconvolution, the emission peak energies were fixed since the observed flakes can be considered as bulk in thickness and the angle-integrated detection does not induce interference in the signal [21]. The deconvoluted field distribution at the highest energy peak of 3.15 eV (*E*_1_ exciton) appears to be uniform over the entire area, whereas for the dielectric resonance mode at 2.45 eV it is most strongly confined at the corners of the flake. Unlike for the *E*_1_ exciton, at the C-exciton energy of 2.78 eV the field is more strongly concentrated at the edges while maintaining a weaker but uniform emission over the remaining flake surface. The A-and B-excitons (1.84 and 2.03 eV) emission is mostly observed for excitation at the corners and edges of the structure. The *E*_*d*_ resonant mode can potentially improve the efficiency of the electron-hole pair generation by the enhanced electromagnetic field contributing to the enhancement of the exciton-related luminescence with the energy levels overlapping or below the *E*_*d*_ peak. In addition to this excitation enhancement, radiative recombination can be accelerated by Purcell effect when the exciton states are electromagnetically coupled with the *E*_*d*_ resonant mode. Besides the incoherent emission, coherently coupled exciton and dielectric modes, or exciton-polariton responses, are also included in the CL signal since the electron beam excitation is broadband [13, 29, 30]. The deconvoluted CL maps clearly indicate that luminescence efficiency of the A and B excitons is improved at the corners due to the existence of the *E*_*d*_ resonant mode [31, 32]. The spectral deconvolution mapping indicates that for this flake the *E*_1_ mode at energy 3.15 eV is not coupled to the dielectric dipole mode, unlike the remaining A and B modes that are possibly coupled to the dielectric dipole mode. We analyzed various flakes with different sizes, which showed different contributions of the dielectric mode coupling to the exciton modes depending on the energy of the dielectric resonance modes, as summarized in Supplementary Material. In short, the decomposed exciton emission peaks become sharper as the spectral overlap with the dielectric mode becomes larger, which could be related to the exciton-polariton resonance. However, since the dielectric resonance peaks are broad, the coupling is not strong enough to show clear peak splits [29, 30]. While these exciton emissions from multilayer MoS_2_ are normally weak in photoluminescence (PL) measurement, although still observable [1], such emission enhancement at the edge is more easily accessible by the high spatial resolution STEM-CL method. We note that the coherently excited exciton-polariton emission in CL is not present in PL measurement.

### 3.2 Shape dependence of existent modes

**Figure 3:**
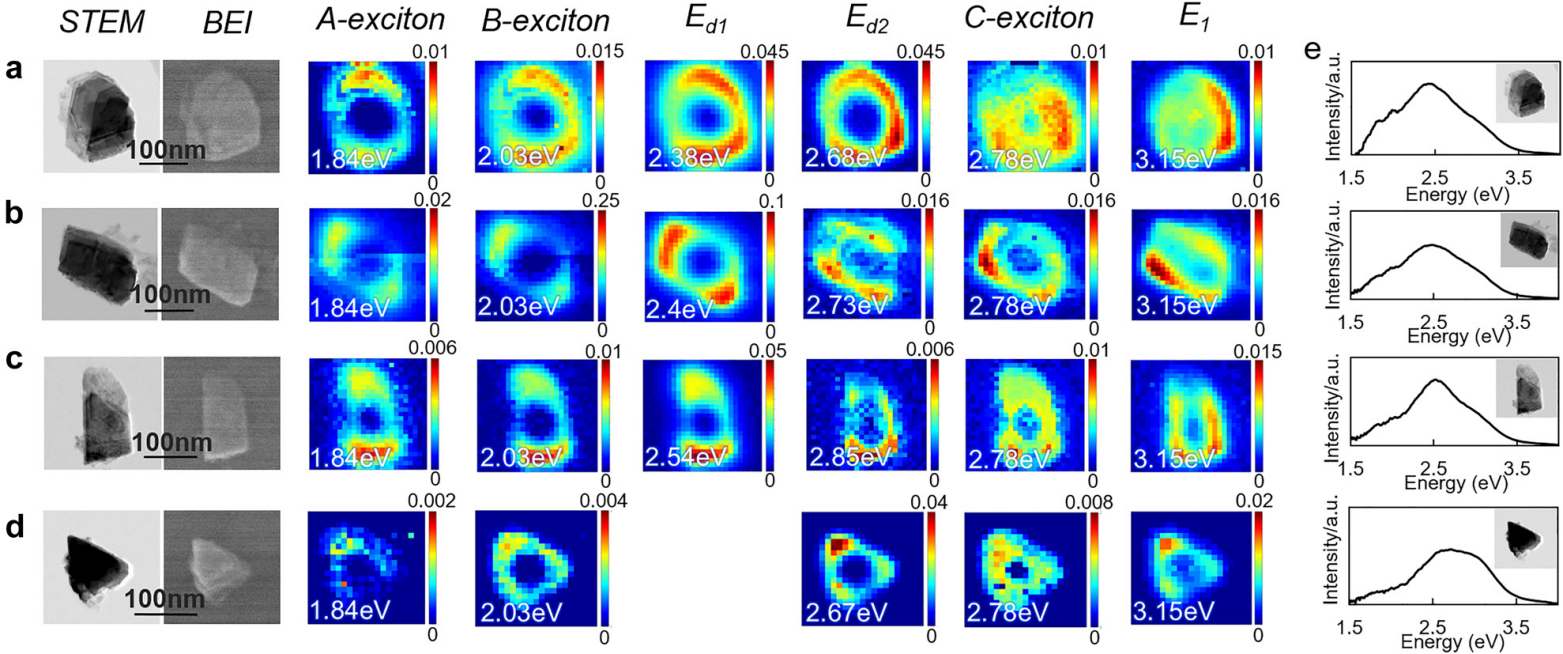
Non-polarized CL maps of individual MoS_2_ nanoflakes with different shapes and sizes. STEM bright field and BEI images, and deconvoluted CL maps experimentally observed for different shapes: (a) oval, (b), (c) rectangular, and (d) triangular nanoflakes, respectively. (e) The experimental CL spectra for the corresponding flakes indicated on the left side. CL signals are collected in all the emission angles without polarization.

We also study the dependence of dielectric resonance modes on different flake shapes. In Figure 3, STEM bright field and backscattered electron images (BEI) confirm the shapes of the flakes under study. From top to bottom of the figure are oval (Figure 3a), rectangular (Figure 3b and c), and triangular shaped flakes (Figure 3d), with lateral sizes between 150 and 250 nm. CL maps are obtained by collecting the emitted signal from all angles. Deconvoluted CL maps are produced by fitting the peaks at fixed energies of the A, B, and C exciton modes as well as at the energies of the dielectric resonance modes which vary according to the flake dimensions. The flake size dependence of the mode is also discussed in the Supplementary Material. Such resonance modes typically exhibit hotspots at the corners or edges of the flakes. Some flakes support more than one dielectric mode while others only one depending on their shapes. For example, in the CL maps of the rectangular flake in Figure 3b, hotspots located at the long-axis edges of the structure correspond to a low energy mode (*E*_*d*_ of 2.4 eV), while hotspots located at the short-axis edge correspond to a higher energy mode (*E*_*d*_ of 2.73 eV), with the energy difference between the two modes arising due to the difference between the length and width of the structure. On the other hand, the smaller triangular flake with almost identical edge lengths supports only one resonance mode energy *E*_*d*_ at 2.67 eV and the field hotspots are located at the structure’s corners. CL maps for the A, B excitons and *E*_1_ emission patterns are found to be influenced by the dielectric modes further providing evidence towards coupled exciton and dielectric modes.

### 3.3 Comparison of experiment and simulation with polarization

**Figure 4:**
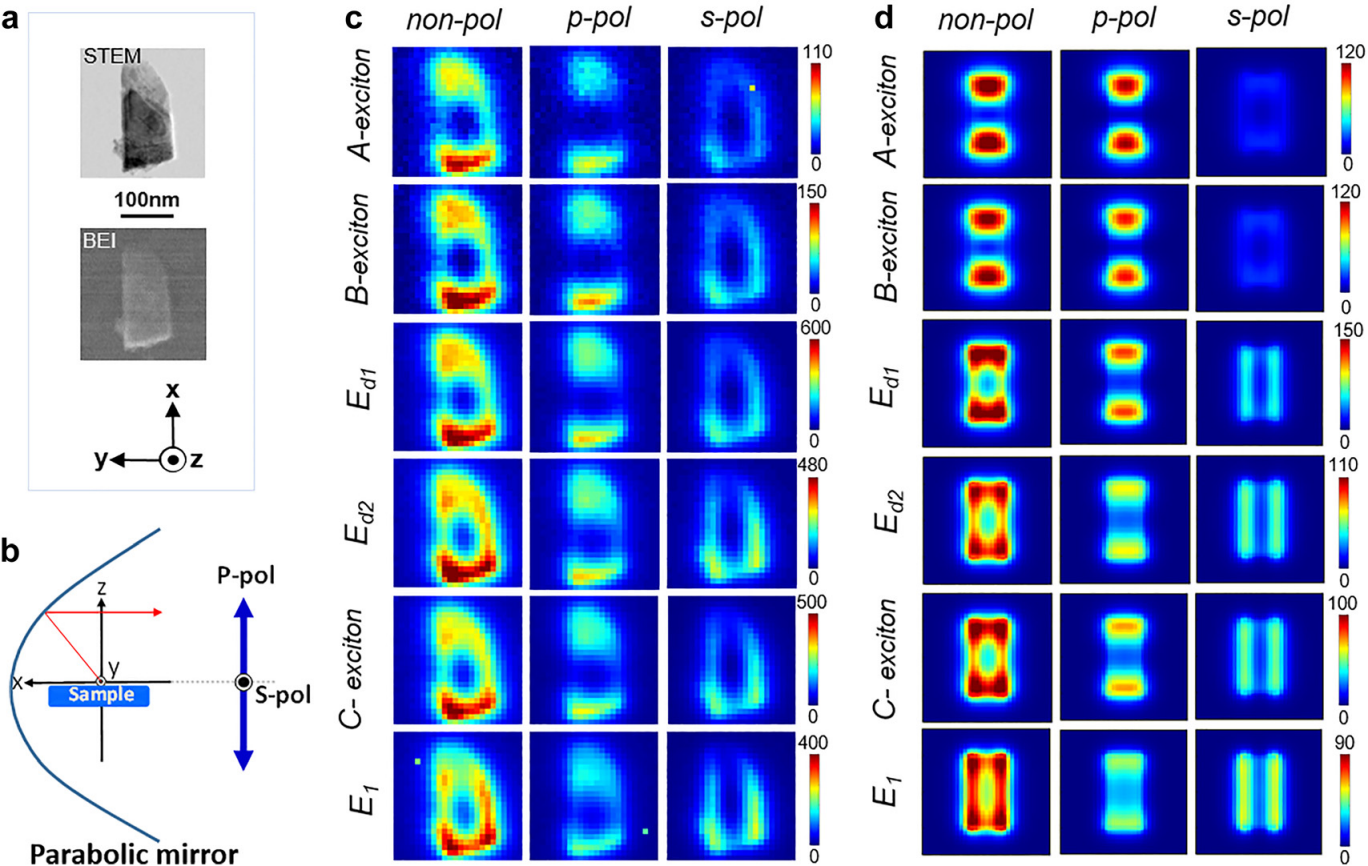
Experiment and BEM simulation CL maps. (a) STEM bright field and BEI images of a rectangular flake with a length of ∼200 nm and width of ∼100 nm. The coordinate fixed at the parabolic mirror is also displayed. (b) Schematic illustration of CL system setting. The CL signal is collected in all the angles. (c) Experimental CL maps without spectral deconvolution for non-, p- and s-polarizations respectively from the left to the right. (d) The corresponding BEM simulation for non-, p- and s-polarizations. For both simulation and experiment results, CL maps display emissions of A-exciton, B-exciton, dielectric modes *E*_*d*1_ and *E*_*d*2,_ *C*-exciton and transition energy *E*_1_, respectively, from top to bottom.

We now perform a more detailed mode analysis with the use of polarization-dependent CL mapping and numerical simulation. A rectangular flake was chosen as a representative shape to demonstrate the polarization dependency of the modes. The polarization dependence is studied by visualizing the radiative electric field with p- and s-polarizations for the rectangular flake shown in Figure 4. Figure 4a shows the STEM image and backscattered electron image (BEI) of the measured rectangular MoS_2_ flake. Figure 4b shows the CL system setting in which the p-polarizer is set parallel to the *x*-*z* plane and the s-polarizer perpendicular to it. We have measured the same flake of Figure 3c with s- and p-polarizations as shown in Figure 4c.

The experimental data is compared with simulation by boundary element method (BEM) [22, 33, 34] for a rectangular MoS_2_ flake with a width of 100 nm, a length of 200 nm, and a thickness of 28 nm, which approximately corresponds to the size of the measured flake. The experimental and simulated spectra obtained from this rectangular flake are included in the Supplementary Material. By comparing the experimental and simulated CL maps in Figure 4c and d, the simulated hotspot distributions are in good agreement. Since the simulation is done in the frequency domain including only coherent responses and still well reproduces the experimental results, the dominant exciton emission should be attributed to the coherently excited exciton polaritons. In the nonpolarized maps where the emission from all the angles is collected, the high energy hotspots are located along the short axis, whereas lower energy hotspots are located along the long axis. To better understand this, we plot the p- and s-polarized maps independently. In the p-polarized simulation maps, the hotspots distribute along the long-axis for all energies, with higher relative intensity for the lower energies. On the other hand, in the s-polarized simulation maps, the hotspots are distributed along the short-axis of the structure for all energies, with stronger relative intensity for the higher energies. Since the photon maps show polarization-dependent patterns with two poles along the long and short axes, these modes can be identified as dipoles. Pure exciton emissions without dielectric coupling would not show such polarization dependence [35]. This result supports that the directions of dipolar momenta of the exciton polaritons are aligned parallel to the long or short axes [36, 37], which are the directions of dipolar momenta of the dielectric resonant modes. When the dipole mode is energetically close to the exciton modes, low energy excitons like A- and B-excitons oscillate in the long axis-direction by coupling with *E*_*d*1_ mode while high energy excitons C- and *E*_1_-excitons have emission spectra polarized along the short-axis by coupling to the *E*_*d*2_ mode.

### 3.4 Angular spectral patterns

**Figure 5:**
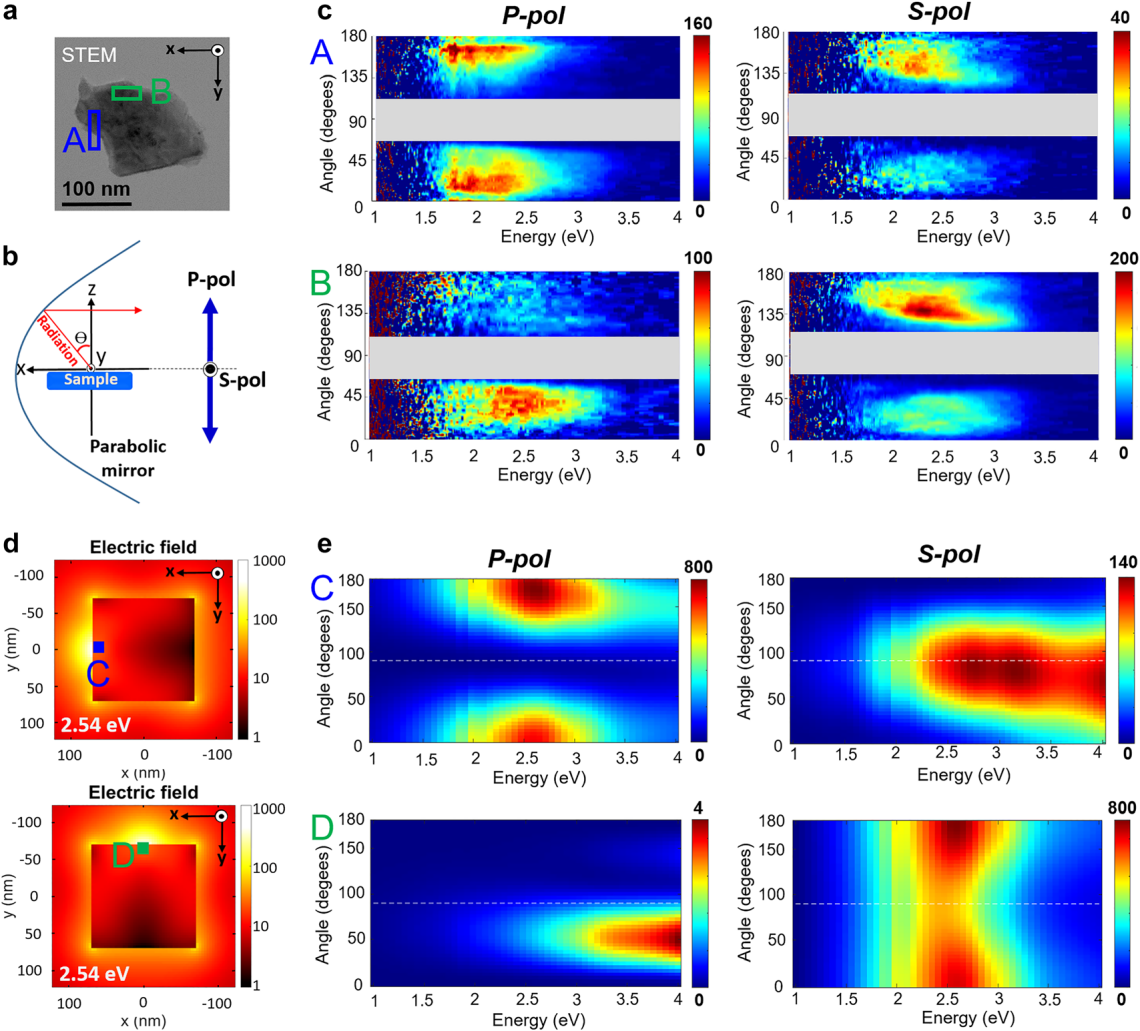
Photon energy and emission angular plots for p and s polarizations. (a) STEM bright-field image of a single MoS_2_ nanoflake with a size of ∼160 nm. (b) Schematic illustration of the detection configuration. Inserting a slit mask parallel to the *z*-axis at *y* = 0 helps obtain the radiated signals at a detection angle ranging from *θ* = 0–180°. (c) Measured emission angular plots for p- and s-polarization of the nanoflake in Figure 5a. The gray-hatched part of the plot around *θ* = 90° is missing due to shadowing by the sample. (d) Calculated time-averaged electric field obtained at *E* = 2.54 eV, on a square flake with the size of 140 nm, for two excitation points C and D. (e) Simulated angle-resolved spectra for the excitation positions C and D with p- and s-polarizations.

Figure 5 shows the angle- and energy-resolved CL spectral plots with the detection angle ranging from 0 to 180°, along the *x*-*z* plane, for a square MoS_2_ flake. The STEM image of the flake is shown in Figure 5a. Angle-resolved CL spectra are shown in panel c for different electron beam positions A and B marked by the red squares in the STEM image. In the angular emission pattern, the signal around *θ* = 90° is missing because of shadowing by the sample and sample holder. It can be seen in the angle-resolved CL spectra that the emission directionality around 1.84 eV mostly follows the dipole emission patterns without the typical Lambertian shape of the non-coupled incoherent emission [35]. This supports that the excitons are polarized by coupling to the flake dipole mode forming exciton polaritons unlike incoherent excitons with random polarization (typically in-plane for TMDs [37]). All these experimental results are qualitatively reproduced by the BEM simulation with a model flake with similar dimensions as the experiment, as shown in Figure 5d and e. We note that only the coherent emission, thus exciton polaritons, based on the dielectric function of the material is considered in the simulation. The simulated angular patterns show the typical emission distributions of an in-plane dipole polarized along the *y*-axis for the excitation position C, and of a dipole along *x*-axis for the excitation position D in the energy range below 3.5 eV. Thus, this polarization- and angular-dependence result again verifies that excitons are coupled to the flake dipole modes although we cannot fully distinguish incoherent exciton emission coupled to the dipole mode and coherent exciton-polariton mode emission in the current measurement.

## 4 Conclusions

We have individually visualized the dielectric resonance and exciton emission modes in isolated MoS_2_ nanoflakes by spectral deconvolution of data obtained with the CL-STEM technique. The MoS_2_ flake exhibits *E*_1_ -exciton band, C-exciton band at 2.78 eV, and the luminescence at lower energies due to B-exciton band (2.03 eV) and A-exciton band at 1.84 eV. The energy and number of dielectric resonance modes of MoS_2_ flakes depend on the flake shape and size, and their emission is dominated by the excitation at the corner and the edge of the structures, where the dielectric field hotspots are located. The electric dipole field distribution and the polarization dependence of the emitted light are confirmed by BEM simulations, showing the coupling of dielectric mode resonance and the exciton modes. In addition, by means of a 4D STEM-CL setup, the angular spectra of the CL emission experimentally revealed the dependence of the emitted field on the emission angle and the field polarization with different excitation positions. The polarization and angle analysis also supports the coupling of the dielectric and exciton modes. The insight about the coupling and enhancement of exciton emission obtained in this study is beneficial for understanding the light-matter interaction as well as for emitter applications such as single-photon systems based on TMDs.

## Supplementary Material

Supplementary Material Details

## Supplementary Material

Effect of particle dimensions on CL spectra, and comparison of experimental and simulated CL spectra for nanoflakes of various sizes. The online version of this article offers supplementary material (https://doi.org/10.1515/nanoph-2021-0643).
