# Supplementary material for: Exciton-dielectric mode coupling in MoS2 nanoflakes visualized by cathodoluminescence
Source: Nanophotonics. 2022 Jan 12;11(9):2129–37. doi: 10.1515/nanoph-2021-0643 (PMC11501478; doi:10.1515/nanoph-2021-0643)
Supplement: Supplementary file 1 — Supplementary Material Details [file j_nanoph-2021-0643_suppl.pdf]

Dung Thi Vu[1], Nikolaos Matthaiakakis[2], Hikaru Saito[3,4], Takumi Sannomiya[1*]

Author addresses

[1] Department of Materials Science and Technology, Tokyo Institute of Technology, 4259 Nagatsuta Midoriku, Yokohama 226-8503, Japan

[2] Theoretical and Physical Chemistry Institute, National Hellenic Research Foundation, NHRF, 48 Vassileos Constantinou Ave., 11635 Athens, Greece

[3] Institute for Materials Chemistry and Engineering, Kyushu University, Fukuoka 816-8580, Japan

[4] Pan-Omics Data-Driven Research Innovation Center, Kyushu University, Fukuoka 816-8580, Japan

* sannomiya.t.aa@m.titech.ac.jp

Supplementary Material for

Exciton-Dielectric Mode Coupling in MoS_2 Nanoflakes Visualized by Cathodoluminescence

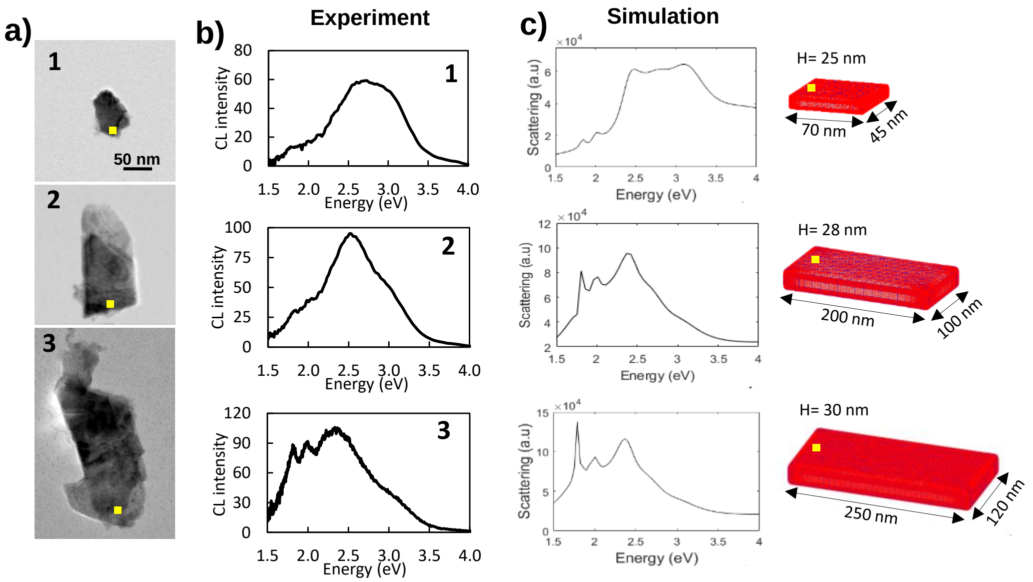

Fig. S1: Effect of particle dimensions on CL spectra. a) STEM images of nanoflakes with various sizes: approximate lateral sizes of (1) 70 nm, (2)200 nm and (3) 250 nm. b) Experimental CL spectra of the flakes of the STEM images in panel a with the excitation positions indicated by yellow squares. The spectra are acquired by integrating all the emission angles. c) Simulated CL spectra and illustrations of the corresponding structures showing flake dimensions (insets on the right side). The structure thicknesses are 25 nm, 28 nm, and 30 nm, respectively. The electron beam positions are indicated by yellow square marks on the structure illustrations.

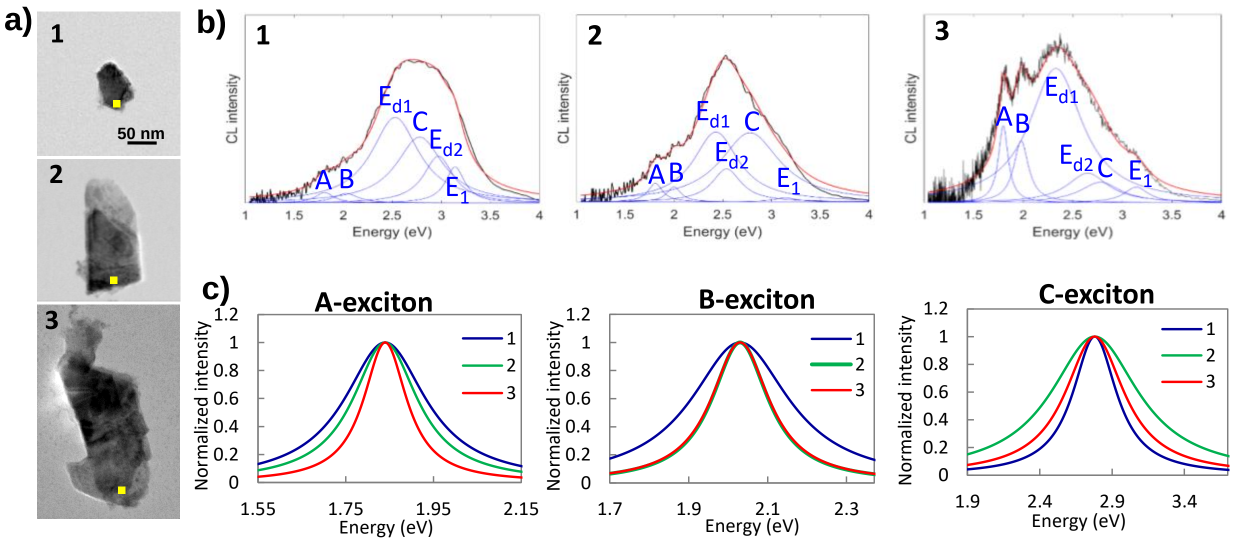

Fig.S2: Sample size dependence of the line widths of the deconvoluted Lorentzian curves for three exciton emissions. The same spectra as Fig.S1 are analyzed. a) STEM images of the measured MoS_2 nanoflakes. b) CL spectra of the selected beam positions indicated by yellow square marks in the corresponding STEM images in (a). c) Deconvoluted Lorentzian curves of individual A-, B-, and C-exciton emissions for different samples shown in (a). The peak widths of all the exciton peaks become sharper as the exciton modes overlap with the dielectric mode (Ed1 or Ed2) resonances. This peak width change could be related to the the exciton-polariton mode line width, which should be more systematically investigated in the future work.
